# Lurasidone and Fluvoxamine Combination in Eating Disorders with Comorbid Obsessive–Compulsive Disorder: Preliminary Evidence from an Observational Study

**DOI:** 10.3390/medsci14010008

**Published:** 2025-12-23

**Authors:** Francesco Monaco, Annarita Vignapiano, Ernesta Panarello, Stefania Landi, Giuseppe Scarano, Giovanna Celia, Giulio Corrivetti, Luca Steardo, Mauro Cozzolino

**Affiliations:** 1 Department of Mental Health, ASL Salerno, 84127 Salerno, Italy; f.monaco@aslsalerno.it (F.M.); annarita.vignapiano@gmail.com (A.V.); stefanialandi173@gmail.com (S.L.); corrivetti@gmail.com (G.C.); 2 European Biomedical Research Institute of Salerno (EBRIS), 84121 Salerno, Italy; 3 Department of Humanities, Philosophy and Education, University of Salerno, 84084 Fisciano, Italy; gscarano@unisa.it (G.S.); mcozzolino@unisa.it (M.C.); 4 Department of Human Sciences, Education and Sport, University of Pegaso, 80100 Naples, Italy; giovanna.celia74@gmail.com; 5 Psychiatric Unit, Department of Health Sciences, University Magna Graecia of Catanzaro, 88100 Catanzaro, Italy; steardo@unicz.it

**Keywords:** anorexia nervosa, obsessive–compulsive disorder, lurasidone, fluvoxamine, exploratory study, Bayesian analysis, missing data

## Abstract

Background: Anorexia nervosa (AN) and obsessive–compulsive disorder (OCD) share core features of cognitive rigidity, anxiety, and altered reward processing. Pharmacological options remain limited, and combined modulation of serotonergic and dopaminergic systems may provide new therapeutic directions. This naturalistic study explored the combined use of lurasidone and fluvoxamine in individuals with restrictive AN (AN-r) and comorbid OCD. Methods: Forty-five female inpatients with AN-r and OCD were followed for six months. Participants received either lurasidone + fluvoxamine (n = 14) or heterogeneous SSRI/antipsychotic regimens (n = 31). Primary outcomes were the *Recovery Assessment Scale (RAS)* and *Body Uneasiness Test Global Severity Index (BUT-GSI)*. Secondary outcomes included the *Eating Disorder Examination-Questionnaire (EDE-Q)* and *Eating Disorder Inventory-3 (EDI-3)*. Bayesian repeated-measures ANOVAs were conducted, reporting *BF*_10_, BFInclusion, and P(M│data) values, with multiple imputation applied to manage missing data. Results: Analyses indicated time-related changes across primary outcomes (RAS and BUT-GSI), with moderate-to-strong evidence (*BF*^10^ = 4.2–18.6) supporting overall improvement during treatment. Secondary and exploratory measures showed weaker or inconsistent trends (*BF*_10_ < 3). No evidence emerged for group-by-time interactions exceeding anecdotal strength. Conclusions: Within the constraints of this small, all-female inpatient cohort, the findings illustrate directional, time-related changes compatible with global rehabilitation effects rather than drug-specific efficacy. The study demonstrates the feasibility—and methodological challenges—of applying Bayesian longitudinal modeling to incomplete clinical datasets. Future randomized or adaptive trials incorporating objective endpoints and data-quality pipelines are warranted to test whether serotonergic–dopaminergic–σ-1 synergy provides genuine clinical benefit in the AN–OCD spectrum.

## 1. Introduction

Eating disorders (EDs) are severe psychiatric conditions characterized by persistent disturbances in eating and related behaviors that impair physical health, psychosocial functioning, and quality of life [1,2]. Their prevalence has risen in recent decades [2], with further increases reported during the COVID-19 pandemic [3]. Among EDs, anorexia nervosa (AN) frequently co-occurs with obsessive–compulsive disorder (OCD). In this study, the term “restrictive anorexia nervosa” (AN-r) refers to the subtype characterized by persistent energy restriction, intense fear of weight gain, and absence of recurrent binge-eating or purging behaviors, as defined by DSM-5 criteria. This presentation often reflects greater cognitive rigidity and compulsive control, features that may overlap with obsessive–compulsive symptomatology.

Patients with AN commonly present with multiple nutritional deficiencies, including vitamins (B1, B6, B12, D, folate) and trace elements (iron, zinc, magnesium, and calcium), which contribute to metabolic, neurocognitive, and affective disturbances [4]. Among these, zinc deficiency appears particularly relevant, as it may exacerbate appetite loss, anxiety, and obsessive traits by altering serotonergic and dopaminergic neurotransmission. Clinical studies suggest that zinc supplementation can facilitate weight restoration and improve mood, supporting its role as a modifiable biological factor in the maintenance of AN [5].

This comorbidity is associated with greater severity, chronicity, and poorer prognosis [6,7,8].

Although the association between OCD and anorexia nervosa is well established, the relationship between OCD and obesity appears to be more complex and heterogeneous. Several epidemiological and clinical studies have suggested that individuals with obesity may display elevated obsessive–compulsive traits, particularly related to food, body image, or ritualized eating behaviors, yet the prevalence of full-threshold OCD among individuals with obesity remains relatively low compared to AN [9]. A meta-analysis by Sharma et al. [9] found that obesity was not among the most common psychiatric comorbidities in OCD, whereas anxiety, depression, and eating disorders were far more prevalent. Conversely, population-based data indicate that OCD and obesity can share underlying neurobiological mechanisms, including dysregulation of cortico-striato-thalamo-cortical circuits, impulsivity–compulsivity spectrum traits, and dopaminergic signaling alterations involved in reward processing [10,11]. Some longitudinal evidence suggests that prior OCD may increase the risk of weight gain or metabolic dysregulation due to medication exposure or behavioral factors (e.g., sedentary rituals, compulsive eating), but these findings remain inconsistent [12]. Taken together, while OCD and obesity may share overlapping pathophysiological pathways and behavioral traits, their direct comorbidity is less robust than that observed between OCD and restrictive eating disorders. This supports the notion that compulsivity may manifest differently across the weight spectrum—restrictive and perfectionistic traits predominating in AN, and impulsive or reward-driven behaviors being more salient in obesity.

While rituals concerning food and weight are intrinsic to AN, OCD in this population is defined by obsessions and compulsions extending beyond eating-related concerns [13]. Clinical evidence suggests that obsessions, more than compulsions, are particularly linked to AN symptomatology [14]. Shared cognitive and temperamental features, such as rigidity and set-shifting difficulties, reinforce the overlap between the two disorders [15]. Evidence-based treatments for OCD and AN remain limited. Cognitive behavioral therapy (CBT) and selective serotonin reuptake inhibitors (SSRIs) are recommended for OCD [16,17], yet many patients fail to respond adequately. In AN, atypical antipsychotics, particularly olanzapine, have been studied, showing modest benefits on weight gain but inconsistent effects on core psychopathology, along with notable metabolic risks [18,19,20,21,22,23,24,25]. Given these limitations, attention has shifted to pharmacological options that might alleviate obsessive–compulsive symptoms without worsening metabolic profiles. Lurasidone, a second-generation antipsychotic with low affinity for histaminergic and muscarinic receptors, combines D_2_/5-HT_2_A antagonism with high-affinity 5-HT_7_ antagonism and 5-HT_1_A partial agonism, properties associated with procognitive, antidepressant, and anxiolytic effects [26,27,28]. Unlike other antipsychotics such as olanzapine, which may support weight restoration but often worsen metabolic parameters and sedation [18,19,25], lurasidone shows a more favorable tolerability profile. Fluvoxamine, beyond its role as an SSRI, acts as a potent σ-1 receptor agonist, an endoplasmic-reticulum chaperone that modulates neuroplasticity, oxidative stress, and ER–mitochondrial signaling [29,30]. The combined use of lurasidone and fluvoxamine is thus grounded in complementary pharmacodynamic mechanisms involving serotonergic and dopaminergic systems, as well as the interplay between 5-HT_7_/σ-1/5-HT_1_A receptors [29,30,31,32]. Preliminary open-label and pilot studies in mood and obsessive–compulsive spectrum disorders suggest that this combination may enhance serotonergic–dopaminergic balance, improving affective rigidity, cognitive control, and depressive symptoms without a significant metabolic burden [31,32].

Activation of σ-1 receptors further enhances neurotrophic and anti-inflammatory pathways, increases BDNF expression, and stabilizes glutamatergic transmission—processes frequently disrupted in anorexia nervosa [33,34]. Through these mechanisms, σ-1 agonism may promote cognitive flexibility, anxiolysis, and appetite regulation, complementing serotonergic modulation and counteracting the rigid, anxiety-driven behaviors typical of AN.

Preliminary case reports suggest that the lurasidone–fluvoxamine combination could be beneficial in patients with AN and comorbid OCD [35,36,37].

In this study, we investigated whether the combined use of lurasidone and fluvoxamine in individuals with restrictive AN (AN-r) and comorbid OCD would be associated with improvements in psychopathological and functional outcomes. By targeting overlapping mechanisms between EDs and OCD, this multimodal strategy may offer a promising therapeutic approach in a population with limited treatment options.

## 2. Materials and Methods

### 2.1. Subjects

Participants were consecutively recruited from the inpatient unit of the Regional Residential Centre for Eating Disorders “Mariconda” (Salerno, Italy). Eligible patients were included if they met the following criteria: (a) DSM-5 diagnosis of AN-r and OCD; (b) Y-BOCS-II > 16; (c) absence of comorbid psychotic disorder, bipolar disorder, or substance abuse; (d) age ≥ 16 years; (e) provision of written informed consent.

Exclusion criteria were (a) severe medical instability requiring intensive care; (b) intellectual disability or cognitive impairment interfering with assessment; (c) for the treatment group, use of pharmacological regimens that could confound outcomes (e.g., other antipsychotics or SSRIs different from lurasidone and fluvoxamine); and (d) refusal or inability to provide informed consent.

A treatment group consisted of patients who received the combination of lurasidone and fluvoxamine in addition to multidisciplinary rehabilitation (psychotherapy and nutritional counseling). A control group was selected from the same inpatient population and included individuals with AN and OCD who received alternative psychopharmacological *regimens* (e.g., SSRIs other than fluvoxamine, or antipsychotics other than lurasidone) together with multidisciplinary rehabilitation. Treatment allocation was based on clinical judgment rather than randomization. The combined lurasidone–fluvoxamine regimen was prescribed to patients showing partial response to SSRI monotherapy or prominent obsessive–affective rigidity, reflecting real-world prescribing practice. Both groups received the same standardized inpatient multidisciplinary program for approximately six months, including psychotherapy, nutritional counseling, and medical monitoring. Adherence was supervised through weekly clinical reviews and medication records, with no significant differences in treatment duration or program participation between groups. Baseline demographic and clinical variables were compared, and any trend-level differences (*p* < 0.10) were included as covariates to mitigate indication bias (Table 1).

All participants had previously undergone at least one course of treatment with SSRI antidepressants prior to inclusion in the study. None of the study participants were past or present drug users, and they did not use drug therapies on a continuous basis, except for sporadic use of analgesics or antipyretics. None of the participants were smokers, both because several were minors and because smoking is not permitted in Regional Residential Centre for Eating Disorders “Mariconda” where they were hospitalized.

Diagnostic confirmation was obtained with the Structured Clinical Interview for DSM-5 (SCID-5). Clinical and demographic data were collected at baseline by trained psychiatrists.

### 2.2. Study Design

This study adopted a naturalistic observational design, reflecting the real-world clinical setting of a specialized residential program for eating disorders. The design was structured as a 2 × 2 repeated-measures framework, incorporating both within-subject and between-subject factors.

The within-subject factor was time, with two assessment points: at baseline (upon admission to the residential unit) and at discharge (after approximately six months of integrated treatment, including psychotherapy, nutritional rehabilitation, and pharmacotherapy). This allowed for the evaluation of longitudinal changes within each participant across the treatment period.

The between-subject factor was group, distinguishing patients who received the combined pharmacological intervention with lurasidone and fluvoxamine (treatment group) from those treated with alternative psychopharmacological regimens (control group). Both groups were embedded within the same multidisciplinary program of residential care, thereby reducing variability due to environmental and therapeutic context.

This configuration enabled us to assess not only the overall changes in symptomatology over time but also whether the trajectories of change differed between the two treatment conditions, as captured by the group × time interaction. In line with the observational nature of the study, allocation to treatment groups was not randomized but derived from the clinical decision-making process.

### 2.3. Assessments

To evaluate treatment outcomes, participants completed a comprehensive battery of validated psychometric instruments administered at two time points: baseline (upon admission to residential care) and discharge (after approximately six months of integrated treatment). The selected measures were chosen to capture multiple domains of functioning, including eating disorder psychopathology, body image disturbance, general psychological distress, and OCD symptoms, thereby providing a multidimensional assessment of clinical change. Treatment response was defined as a clinically meaningful improvement across primary outcome domains, operationalized by a reduction of at least 20% in baseline scores on disorder-specific psychometric measures (e.g., Y-BOCS for OCD symptoms, EDI-3 for eating-disorder traits, and SCL-90-R subscales for depressive and distress symptoms), or by a positive change corresponding to a moderate or greater effect size in the Bayesian repeated-measures model.

- **Eating Disorder Inventory-3 (EDI-3).**

The EDI-3 is a widely used self-report instrument designed to assess psychological traits and behavioral dimensions relevant to eating disorders [38]. It includes 91 items grouped into 12 primary scales, such as Drive for Thinness, Bulimia, and Body Dissatisfaction, along with composite indices reflecting eating disorder risk, ineffectiveness, interpersonal insecurity, and emotional dysregulation. The instrument has demonstrated robust psychometric properties, with internal consistency ranging from 0.79 to 0.97 across subscales and strong construct validity in both clinical and non-clinical populations.

- **Eating Disorder Questionnaire (EDE-Q).**

The EDE-Q is a 28-item self-report questionnaire, adapted from the semi-structured interview, the Eating Disorder Examination (EDE). The questionnaire is designed to assess the range, frequency and severity of behaviors associated with a diagnosis of an eating disorder. It is categorized into 4 subscales (Restraint, Eating Concern, Shape Concern and Weight Concern) and an overall global score, with a higher score indicating more problematic eating difficulties [39].

- **Body Uneasiness Test (BUT).**

The Body Uneasiness Test (BUT) comprises two complementary sections: BUT-A, which measures overall body-image dissatisfaction and related psychopathological dimensions (Weight Phobia, Body Image Concerns, Avoidance, Compulsive Self-Monitoring, and Depersonalization), and BUT-B, which assesses dissatisfaction with specific body parts and functions. Subscale scores range from 0 to 4, with higher values indicating greater disturbance. In this study, both the Global Severity Index (BUT-GSI) and relevant BUT-A subscales were analyzed to capture body-related anxiety and cognitive–affective components of body image disturbance [40]. The inclusion of both sections allowed differentiation between general body uneasiness and specific perceptual–attitudinal concerns, which are clinically relevant in AN. The BUT has been shown to effectively discriminate between patients with eating disorders and healthy controls, with Cronbach’s α coefficients above 0.80.

- **Symptom Checklist-90-Revised (SCL-90-R).**

The SCL-90-R is a multidimensional inventory designed to evaluate a wide spectrum of psychological symptoms and distress [41]. Composed of 90 items rated on a 5-point Likert scale, it covers nine symptom dimensions: Somatization, Obsessive–Compulsive, Interpersonal Sensitivity, Depression, Anxiety, Hostility, Phobic Anxiety, Paranoid Ideation, and Psychoticism. The Global Severity Index (GSI) provides an overall measure of symptom burden, integrating intensity and frequency. The scale is extensively validated and widely used in both clinical practice and research to monitor changes over time.

- **Yale–Brown Obsessive–Compulsive Scale, Second Edition (Y-BOCS-II).**

The Y-BOCS-II is a semi-structured clinician-administered interview designed to assess the severity of obsessive–compulsive symptoms [42]. It includes 10 core items evaluating time occupied by obsessions and compulsions, associated distress, interference, resistance, and perceived control. Scores range from 0 to 40, with higher scores indicating greater severity. The revised version enhances sensitivity to treatment-related change and demonstrates excellent inter-rater reliability, test–retest reliability, and convergent validity.

- **Exercise Dependence Scale (EDS).**

The EDS assesses problematic patterns of physical activity conceptualized as a behavioral addiction [43]. Adapted from DSM-IV substance dependence criteria, it consists of 21 items rated on a six-point Likert scale (1 = never to 6 = always), covering seven domains: tolerance, withdrawal, intention effects, lack of control, time investment, reduction in other activities, and continuation despite negative consequences. The EDS provides a validated framework for evaluating exercise-related compulsivity in clinical populations. The Recovery Assessment Scale (RAS) was used as a primary outcome to evaluate subjective recovery and perceived autonomy in daily functioning. It consists of 24 items rated on a 5-point Likert scale, with higher scores reflecting greater psychological recovery and empowerment. The RAS was chosen for its sensitivity to functional and attitudinal improvement during multidisciplinary rehabilitation.

- The Life Skills Profile (LSP) assessed functional capacity in interpersonal relationships, communication, and community adaptation. Higher scores indicate better psychosocial functioning and are considered predictive of global outcome in chronic psychiatric conditions.
- The Clinical Impairment Assessment (CIA) measured the psychosocial impact and distress associated with eating-disorder symptoms. It provides a global score reflecting impairment in mood, social, and cognitive functioning, with higher scores indicating greater functional disability.

Together, these instruments offered a robust multidimensional evaluation of eating disorder symptoms, OCD severity, general psychopathology, and body image disturbance. Their combined use enabled a comprehensive assessment of treatment effects across clinically relevant domains. Although several instruments (EDI, EDE-Q, CIA, BUT) assess overlapping aspects of eating-disorder psychopathology, they were selected to capture complementary dimensions—trait versus state features, cognitive versus behavioral symptoms, and body-image versus functional impairment components. This multidimensional approach allowed cross-validation of findings across independent but convergent measures, thereby strengthening construct validity.

### 2.4. Analytic Approach—Bayesian Repeated-Measures ANOVA

Bayesian repeated-measures ANOVAs were conducted using the *BayesFactor* package [44] (Morey & Rouder, 2018) implemented in jamovi [45]. Models were estimated under the default Jeffreys–Zellner–Siow (JZS) priors [46], specifying a Cauchy distribution centered at zero with scale parameter *r* = 0.5 for fixed effects. Random effects (subjects) were assigned non-informative priors, representing a weakly informative assumption that most effects are small while still allowing for moderate-to-large deviations. For each outcome, we compared a pre-specified hierarchy of competing models:

1. Null model: random intercept for subject only (accounts for within-subject dependence).
2. Group model: between-subjects main effect of group.
3. Time model: within-subjects main effect of time.
4. Additive model: group + time (no interaction).
5. Full model: group + time + group × time (interaction).

All models included a random intercept for subject to accommodate repeated measures. Evidence for each model was summarized using Bayes Factors (*BF*_10_*:* evidence for the alternative model vs. the null), inclusion Bayes Factors (*BFInclusion)* for specific effects, and posterior model probabilities *P(M│data)*. Interpretation followed Jeffreys’ [47] evidence scale: *BF*_10_ = 1–3 (*anecdotal*), 3–10 (*moderate*), 10–30 (*strong*), 30–100 (*very strong*), and >100 (*decisive*). Pre-specified post hoc pairwise contrasts were performed where relevant and adjusted for multiplicity using the Bayesian Westfall–Johnson–Utts procedure [48] as implemented in jamovi. Both uncorrected and corrected Bayes Factors are reported for transparency to Supplementary Table S1.

### 2.5. Missing Data and Imputation Strategy

Because several outcomes exhibited non-trivial proportions of missing data—particularly at follow-up—we implemented a fully explicit, two-pronged non-parametric imputation strategy with diagnostic evaluation rather than relying on single, ad hoc substitution methods. Specifically, missing values were imputed using two random-forest–based algorithms, *MissForest* [49] and *missRanger* [50]. These methods were chosen because they handle mixed variable types, preserve non-linear relationships, and provide internal diagnostics (out-of-bag error estimates or variance-explained metrics) that assist in assessing imputation fidelity. For each continuous variable MissForest produced an out-of-bag (OOB) mean squared error (MSE); missRanger produced an analogous metric (proportion of variance unexplained, PVU, equal to 1 − R^2^ in the model predicting that variable). Lower MSE (MissForest) and lower PVU (missRanger) indicate better reconstruction quality. We considered these diagnostics jointly when judging imputation reliability.

For transparency, a concise summary of missingness and imputation diagnostics (MissForest OOB-MSE and missRanger PVU) is reported in the main text (Table 2); full outputs and algorithm parameters are provided in Supplementary Table S1.

Across all psychometric entries included in the imputation procedure, 313 missing values were imputed (imputation log). Considering the principal clinical scales/timepoints summarized in Table 2 (20 variable × timepoint entries reported in the main text), 277 of 900 expected observations were imputed (277/900 = 30.8%). The difference (313 vs. 277) reflects additional subscales and item-level entries included in the complete imputation procedure (see Supplementary Table S1 for the exhaustive list). To guide interpretability we pre-specified conservative thresholds for imputation acceptability: MissForest OOB-MSE < 1 and missRanger PVU < 0.30 across timepoints to classify an outcome as ‘Robust’. Outcomes not meeting these thresholds were classified as ‘Mixed/Cautious’ or ‘Exploratory’ (Table 2). For inferential claims, we required both acceptable reconstruction diagnostics and concordant inferential results across the two imputation methods; otherwise, outcomes are reported as exploratory and hypothesis-generating.

Robust: *MissForest* OOB-MSE < 1 and *missRanger* PVU < 0.30 at both time points, with concordant Bayesian results (same favored model family or similar Bayes-factor magnitude) across imputations.

- Mixed/Cautious: one imputation method met the criterion while the other was borderline, or OOB-MSE/PVU values worsened at follow-up.
- Exploratory: OOB-MSE or PVU was large for at least one method (e.g., OOB-MSE ≫ variable SD or PVU ≈ 1) or there was clear discordance between methods and imputation-specific inferential results.

Both algorithms rely on iterative random-forest models to predict missing entries from observed variables without assuming parametric distributions or Bayesian priors. Default parameters were used (500 trees, 10 iterations, *mtry* ≈ *p*/3). *MissForest* reports OOB-MSE as an internal quality index, whereas *missRanger* provides PVU. These thresholds were intentionally pragmatic and conservative, chosen to favor reproducibility and avoid overstating evidence based on unstable estimates.

### 2.6. Outcome Hierarchy

In accordance with the exploratory design, outcome measures were pre-classified to prevent overlap between primary, secondary, and exploratory domains. Primary outcomes included the Recovery Assessment Scale (RAS) and the Body Uneasiness Test—Global Severity Index (BUT-GSI), representing the most reliable and clinically central indicators of psychological recovery and body-image distress. Secondary outcomes comprised the Eating Disorder Examination Questionnaire (EDE-Q) and the Eating Disorder Inventory-3 (EDI-3), which captured broader cognitive–behavioral eating-disorder traits but were characterized by higher missingness. Exploratory outcomes encompassed all remaining scales (e.g., SCL-90-R, CIA, EDS, Y-BOCS-II, and subscale analyses) used to generate preliminary hypotheses about comorbid symptom dimensions. This hierarchy was maintained in the presentation and interpretation of results, with primary outcomes emphasized in the main text and secondary/exploratory findings discussed as hypothesis-generating only.

## 3. Results

Participants were subdivided into two groups: the treatment group included 14 women and the control group 31 women. The mean age in the treatment group was 18.9 years (SD = 3.45), whereas in the control group it was 19.6 years (SD = 7.15); age and data were missing for three participants (Table 1). The treatment group included 8 adults and 6 adolescents, while the control group included 12 adults and 16 adolescents (with three missing values for age category). The average number of years of schooling was 2.64 (SD = 0.633) in the treatment group and 2.40 (SD = 0.645) in the control group. To address missing data, two independent non-parametric imputation procedures were applied (MissForest and MissRanger) and Bayesian repeated-measures ANOVAs were then performed on the resulting imputed datasets (Table 3). Variables were classified as robust, mixed/cautious, or exploratory on the basis of: (1) diagnostic indices of imputation quality (MissForest OOB MSE and MissRanger PVU/1 − R^2^), and (2) concordance of inferential results (Bayes Factors and posterior model probabilities) across the two imputation methods. Numerical diagnostics and classification rules are reported in Table 2; full model outputs are reported in Table S1 supplementary. Overall imputation burden. Across the principal clinical scales/timepoints shown in Table 2 (20 variable-timepoint entries), 277 of 900 observations were imputed (30.8%); across the full imputation log used for analysis (including additional subscales and item-level variables) 313 missing values were imputed (imputation log). Rationale for interpretability despite missingness. When both imputation diagnostics satisfied the pre-specified thresholds (MissForest OOB-MSE < 1 and missRanger PVU < 0.30 at both timepoints) and Bayesian inferences were concordant across imputations, we treated temporal effects as cautiously interpretable and labeled those outcomes ‘Robust’. For measures with elevated reconstruction error or discordant inferential outcomes (labeled ‘Mixed/Cautious’ or ‘Exploratory’), results are presented as hypothesis-generating and interpreted with clear caveats. Presentation of Bayes factors. In the main Results we summarize Bayes Factors and posterior probabilities narratively using Jeffreys’ descriptors (e.g., anecdotal, moderate, strong, very strong, decisive). Numeric *BF* matrices, posterior model probability tables, and corrected vs. uncorrected *BF* values have been moved to the Supplementary Material (Table S1).

### 3.1. Robust/Relatively Stable Outcomes

The Recovery Assessment Scale (RAS), which indexes subjective psychological recovery, showed a consistent time effect in both imputation analyses. Under MissForest the best-supported model corresponded to a very strong/decisive evidence for a time effect (*BF*_10_ = 7.15 × 10^9^, P(M│data) = 0.604) and under MissRanger results were concordant (*BF*_10_ = 1.68 × 10^12^, P(M│data) = 0.558). MissForest reported modest OOB MSE values at baseline and follow-up (T0 = 0.283; T1 = 0.222), whereas MissRanger reported larger PVU values; accordingly RAS is classified as mixed (see Table 2).

The Body Uneasiness Test—Global Severity Index (BUT-GSI) demonstrated low imputation error in both procedures (MissForest OOB MSE T0 = 0.355, T1 = 0.537; MissRanger PVU T0 = 0.0952, T1 = 0.2145) and was classified as robust. Bayesian model comparisons consistently supported a very strong/decisive evidence for a time effect (MissForest: *BF*_10_ ≈ 5.20 × 10^3^, MissRanger: *BF*_10_ ≈ 3.68 × 10^3^).

The BUT Positive Symptom Distress Index (BUT-PSDI) likewise indicated a strong evidence for a time effect in both imputations (MissForest *BF*_10_ ≈ 2.10 × 10^2^; MissRanger *BF*_10_ ≈ 9.02 × 10^1^). MissForest OOB MSE was low at baseline (T0 = 0.251) but increased at follow-up (T1 = 0.708); MissRanger PVU showed acceptable but increased values at T1 (T0 = 0.1044; T1 = 0.2764). Given this pattern, BUT-PSDI is classified as mixed-to-cautious: the inferential evidence for a reduction in symptom intensity over time is present, but interpretative confidence is tempered by the increased imputation error at follow-up.

### 3.2. Exploratory Outcomes

The global score of the Eating Disorder Examination Questionnaire (EDE-Q) exhibited marked heterogeneity in imputation diagnostics. MissForest reported a very large OOB MSE at baseline (T0 = 170.531) and a much smaller value at follow-up (T1 = 0.701); MissRanger reported high PVU at baseline (T0 = 1.1534) and improved PVU at follow-up (T1 = 0.1850). Consequently, the EDE-Q was classified as exploratory. Bayesian comparisons provided moderate to strong evidence for a time effect (MissForest *BF*_10_ ≈ 31.0; MissRanger *BF*_10_ ≈ 14.2), but given the substantial uncertainty surrounding baseline imputations these results are reported as preliminary and hypothesis-generating only.

Several other secondary measures and subscales displayed large OOB MSE or high PVU values in one or both imputation procedures and/or produced inconsistent Bayesian evidence across imputations. For these variables the Bayes Factors varied between anecdotal and moderate levels of evidence depending on the imputation method; accordingly, all such outcomes are not presented. Complete imputation diagnostics for all variables kept are provided in Table 2 so that readers can evaluate the relative reliability of each imputed outcome.

### 3.3. Clinical Interpretation

From a clinical standpoint, the pattern of findings across the main outcomes suggests a generalized improvement over time, with variability in robustness and interpretative confidence depending on each construct.

**Figure 1:**
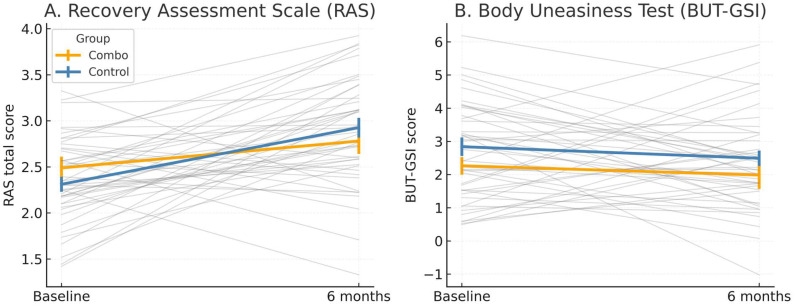
Trajectories of clinical change from baseline to six months. (**A**) Gray lines represent individual participant trajectories, while colored lines depict group means ± standard error (orange = Lugreatwridone + Fluvoxamine; blue = Control). Imputed data were generated using a non-parametric MissForest algorithm based on available psychometric and demographic predictors. At baseline (T_0_), all participants contributed data (n = 45; 14 Combo + 31 Control). At six months (T_1_), complete observations were available for 36 participants (n = 11 Combo + 25 Control), with missing values replaced by imputed estimates. Error bars reflect within-subject standard error. These findings should be interpreted as exploratory, given the small, all-female sample and naturalistic design. (**B**) Gray lines represent individual trajectories; colored lines indicate group means ± standard error (orange = Lurasidone + Fluvoxamine; blue = Control). Data were imputed using the same MissForest multiple-imputation procedure applied across psychometric outcomes. At baseline (T_0_), n = 45 participants contributed data (14 Combo, 31 Control); at six months (T_1_), n = 36 (11 Combo, 25 Control). Error bars denote within-subject standard error. Results are illustrative of directionality rather than confirmatory, owing to sample size and missingness constraints.

For the Recovery Assessment Scale (RAS)—which captures subjective psychological recovery through domains such as hope, empowerment, and self-agency—both imputation procedures indicated a consistent time effect, with substantial Bayesian support. This pattern implies that participants reported an enhanced sense of psychological recovery from baseline to follow-up. The concordance between MissForest and MissRanger imputations reinforces the likelihood that this improvement reflects a true temporal change rather than an artifact of data reconstruction. However, given the modest imputation error, these findings should still be regarded as tentative indicators of preliminary association rather than confirmatory evidence (Figure 1).

Regarding the Body Uneasiness Test—Global Severity Index (BUT-GSI), both imputations showed low error and stable Bayesian evidence for a main time effect, with occasional support for additive effects of group and time. Clinically, this corresponds to a reduction in body-related distress across the observation period, suggesting that both treatment conditions may contribute to an alleviation of body uneasiness. The convergence of the two imputation methods, coupled with low reconstruction error, indicates that this effect is relatively robust, though caution remains warranted due to the non-randomized design and small sample.

The BUT Positive Symptom Distress Index (BUT-PSDI) displayed a consistent time effect in both imputations, supporting a decrease in the intensity of body-related distress symptoms over time. However, the increase in imputation error at follow-up (particularly under MissForest) reduces confidence in the precision of this estimate. Consequently, while the trend toward improvement is clinically plausible and directionally consistent with other indicators of body-image relief, these results should be interpreted with caution, acknowledging their mixed-to-cautious classification (Figure 1). Figure 1 display individual and mean trajectories for the two primary outcomes. Both panels include imputed estimates (MissForest algorithm) and show the number of participants contributing observed data at each time point.

Finally, the Eating Disorder Examination Questionnaire (EDE-Q) showed the most variable imputation diagnostics, particularly due to a very large baseline error. Although Bayesian analyses yielded moderate evidence for a main time effect—suggesting a potential reduction in cognitive-behavioral eating disorder symptoms—the instability of the imputed baseline data precludes firm conclusions. Clinically, the observed pattern may still indicate meaningful improvement in eating disorder psychopathology over time, yet such interpretation should be viewed as hypothesis-generating rather than confirmatory.

Overall, these findings converge toward a pattern of general improvement over time, with the most consistent and reliable evidence emerging for psychological recovery (RAS) and body uneasiness reduction (BUT-GSI). Nevertheless, given the observational nature of the study, small sample size, and residual uncertainty from imputation, all interpretations must remain cautious and exploratory, pending confirmation through larger and randomized investigations.

### 3.4. Overall Synthesis and Cautious Interpretation

Taken together, a subset of outcomes related to subjective recovery and body-image distress (RAS and BUT measures) exhibited consistent temporal improvement and reasonably concordant inferential support across both MissForest and MissRanger imputations; these measures are therefore reported as the most interpretable results of the study (Table 2). Other measures, notably the EDE-Q and several secondary scales, were characterized by elevated imputation uncertainty at one or both timepoints and by less stable Bayes Factors; results for these outcomes are therefore designated exploratory and interpreted with caution.

All inferential statements are presented without claims of causality. Given the modest sample size and the variable pattern of missingness, we emphasize that these findings are preliminary: they should be confirmed in larger, prospectively collected samples with pre-specified handling of missing data.

## 4. Discussion

The present exploratory study aimed to examine potential temporal and group-related changes associated with a combined lurasidone–fluvoxamine regimen in individuals with restrictive anorexia nervosa (AN-r) and comorbid obsessive–compulsive disorder (OCD). This investigation was conceived as a preliminary, hypothesis-generating effort rather than a test of efficacy, given the small sample, non-randomized design, and incomplete follow-up data.

Although Bayesian repeated-measures ANOVA models revealed consistent time effects in the primary outcomes (RAS and BUT-GSI), secondary and exploratory measures (EDE-Q, EDI, CIA, Y-BOCS) yielded less stable or inconclusive evidence. The robustness of these findings is limited by the extent of missing data and the uncertainty introduced by imputation. Although the assumption of data Missing Completely at Random (MCAR) was not statistically refuted (*p* = 0.192), missingness ranged from 20% to over 60% across key variables (e.g., RAS, EDE-Q, EDI). The magnitude of imputation error (PVU = 0.56–1.15 for RAS and EDE-Q) further indicates that the reconstructed values are only approximate representations of the original dataset. Consequently, both the magnitude and direction of the estimated effects should be regarded as unstable and non-generalizable [49,51,52].

Within these methodological boundaries, the most consistent pattern observed was a general improvement over time, largely independent of treatment group. Strong inclusion Bayes Factors (*BF* > 100 for most time models) suggest moderate-to-strong evidence for within-subject change, particularly in global distress (SCL-GSI), body image discomfort (BUT-GSI), and perceived recovery (RAS). However, such improvements are likely to reflect the multimodal rehabilitation context—comprising psychotherapy, nutritional support, and structured social re-engagement—rather than pharmacological effects per se [1,7].

In contrast, no decisive evidence emerged for main group effects or group × time interactions. Posterior odds and inclusion Bayes Factors for these terms consistently fell below conventional thresholds (*BF* < 1), suggesting that any apparent group differences between the lurasidone–fluvoxamine and control conditions may represent sampling variability or measurement noise rather than true pharmacodynamic divergence. Considering the limited sample, absence of randomization, and incomplete longitudinal data, it is plausible that the observed temporal improvements stem from nonspecific therapeutic influences or regression to the mean [46]. Nonetheless, certain exploratory trends may hold theoretical interest. The consistent time-linked reductions in distress and body uneasiness are compatible with, though far from confirming, the hypothesis that a combined modulation of serotonergic and dopaminergic signaling could support emotional regulation and cognitive flexibility [26,27,30]. These mechanisms might contribute to the gradual attenuation of rigid and perfectionistic cognitive patterns characteristic of AN–OCD comorbidity [6,53]. However, given the exploratory design and data limitations, no causal inferences should be drawn at this stage. Taken together, the findings underscore how data integrity, sample size, and analytic assumptions critically shape Bayesian inference. While the Bayesian framework offers conceptual advantages—particularly in quantifying uncertainty and accommodating small samples [47,48]—its interpretive value diminishes when imputation variance exceeds explained variance. Consequently, this study should be regarded primarily as a feasibility and proof-of-concept analysis, illustrating methodological applicability rather than demonstrating clinical efficacy. While the present findings provide preliminary support for the mechanistic plausibility and clinical feasibility of combining lurasidone with fluvoxamine in AN–OCD, their interpretative value remains limited by the small, all-female sample and the naturalistic, non-randomized design. Accordingly, the primary implication of this study is not to advocate further exploratory analyses, but rather to encourage the design of future randomized controlled or adaptive trials capable of disentangling drug-specific effects from general rehabilitation outcomes. Such studies should integrate longitudinal, multimodal, and mechanistic measures to clarify the contribution of serotonergic, dopaminergic, and sigma-1 receptor pathways to treatment response.

### Limitations

This study has several important limitations. The small, all-female sample, naturalistic design, and non-randomized treatment allocation restrict the strength of inference regarding pharmacological effects [35,36,37]. Missing data and reliance on multiple imputation introduce additional uncertainty, while the heterogeneity of the control group limits comparability [38]. The exclusive use of self-report instruments, without neurobiological or behavioral validation, further constrains measurement precision. From a methodological standpoint, the analysis was limited by incomplete data, modest statistical power, and a relatively short follow-up interval. Although Bayesian longitudinal modeling provided a flexible analytic framework, it cannot fully offset sampling bias or data sparsity inherent to small naturalistic cohorts [39,40]. Regarding clinical generalizability, the findings apply primarily to a highly selected inpatient female population undergoing multidisciplinary rehabilitation. Improvements observed are therefore more plausibly attributed to global rehabilitation effects than to the specific pharmacological action of the lurasidone–fluvoxamine combination. Future randomized or adaptive trials, integrating objective physiological and behavioral endpoints and ensuring robust data quality pipelines, are needed to confirm these preliminary findings and their translational relevance [41,42,54,55].

## 5. Conclusions

Given the extent of data loss and the uncertainty introduced by imputation, the present analysis should be regarded as a pilot, hypothesis-generating investigation rather than as evidence of clinical efficacy. The observed temporal improvements across psychometric domains are more plausibly explained by the global rehabilitation effects of a structured in-patient program than by the specific pharmacological action of the lurasidone–fluvoxamine combination. Despite these constraints, this study demonstrates the feasibility and methodological challenges of applying Bayesian longitudinal models to small and incomplete clinical datasets. The experience underscores the need for systematic data collection, standardized follow-up intervals, and transparent analytic workflows in future investigations. The primary implication of this work is the need for well-powered, randomized or adaptive clinical trials to determine whether dopaminergic–serotonergic–sigma-1 synergy can provide a potential benefit in the AN–OCD spectrum. Future trials should integrate multi-imputation benchmarking, data quality assurance pipelines, and objective physiological and behavioral endpoints, ensuring that computational inference remains aligned with clinical reality.

## Figures and Tables

**Table 1:** Descriptive analysis of all samples, median rehabilitation duration = 24 weeks (IQR 23–26).

Variable	Treatment Group (n = 14)	Control Group (n = 31)
Age (years)	18.9 (±3.45)	28 (±7.15)
Adults (n, %)	8 (19%)	12 (28.6%)
Adolescents (n, %)	6 (14.3%)	16 (38.1%)
Primary school certificate (n, %)	0 (0%)	1 (2.6%)
Middle school certificate (n, %)	6 (15.4%)	14 (35.9%)
High school diploma (n, %)	7 (17.9%)	9 (23.1%)
Bachelor’s degree (n, %)	1 (2.6%)	1 (2.6%)

**Table 2:** Imputation diagnostics comparing MissForest (out-of-bag mean squared error) and MissRanger (proportion of falsely predicted values).

Variable	Missing n (T0/T1)	% Missing (T0/T1)	MissForest OOB-MSE (T0/T1)	MissRanger PVU (T0/T1)	Classification
RAS	15–36	33.3%/80.0%	0.283/0.222	0.5953/0.5655	Mixed
BUT—GSI	8–21	17.8%/46.7%	0.355/0.537	0.0952/0.2145	Robust
BUT—PSDI	9–21	20.0%/46.7%	0.251/0.708	0.1044/0.2764	Mixed-to-cautious
EDE-Q (global)	23/19	51.1%/42.2%	170.531/0.701	1.1534/0.1850	Exploratory

Note: percentages are calculated on the total sample (N = 45) for each time point. MissForest OOB-MSE and missRanger PVU are reported for each time point when available.

**Table 3:** Bayesian repeated-measures ANOVA results for primary clinical variables under MissForest and MissRanger imputations.

Measure	Best Model (MissForest)	*BF*_10_ (MissForest)	*P(M/data)* (MissForest)	Best Model (MissRanger)	*BF*_10_ (MissRanger)	*P(M/data)* (MissRanger)	Interpretation (Jeffreys)
Recovery Assessment Scale (RAS)	time	7.15 × 10^9^	0.604	time	1.68 × 10^12^	0.558	Very strong → decisive evidence for a time effect (improvement over time).
BUT—GSI	time	5.20 × 10^3^	0.517	time	3.68 × 10^3^	0.524	Very strong evidence for a time effect; additive models (group + time) also supported in some configurations.
BUT—PSDI	time	2.10 × 10^2^	0.590	time	9.02 × 10^1^	0.610	Strong evidence for a time effect (reduction in symptom intensity).
EDE-Q (global)	time	3.10 × 10^1^	0.650	time	1.42 × 10^1^	0.555	Moderate to strong evidence for a time effect; additive model also has some support.

Note. *BF*_10_ values represent the Bayes Factor in favor of the alternative hypothesis (H_1_) over the null (H_0_); P(M|data) indicates posterior model probability. Interpretive thresholds follow Jeffreys [47]: 1–3 = anecdotal evidence, 3–10 = moderate, 10–30 = strong, 30–100 = very strong, > 100 = decisive evidence. Concordance across imputation methods supports the robustness of observed time effects, while discrepancies are to be interpreted cautiously.

## Data Availability

The original contributions presented in this study are included in the article/Supplementary Material. Further inquiries can be directed to the corresponding author.

## Supplementary Materials

The following supporting information can be downloaded at https://www.mdpi.com/article/10.3390/medsci14010008/s1, Table S1: Imputation diagnostics comparing MissForest (out-of-bag mean squared error) and MissRanger (proportion of falsely predicted values.
