# Supplementary material for: Lurasidone and Fluvoxamine Combination in Eating Disorders with Comorbid Obsessive–Compulsive Disorder: Preliminary Evidence from an Observational Study
Source: Med Sci (Basel). 2025 Dec 23;14(1):8. doi: 10.3390/medsci14010008 (PMC12821398; doi:10.3390/medsci14010008)
Supplement: Supplementary file 1 [file medsci-14-00008-s001.zip › medsci-3880100-supplementary.pdf]

Table S1. Imputation diagnostics comparing MissForest (out-of-bag mean squared error) and MissRanger (proportion of falsely predicted values).

Variable	Missing n (T0/T1)	% missing (T0/T1)	MissForest OOB-MSE (T0 / T1)	missRanger PVU (T0/T1)	Classificazione
EDI	6-26	13.3% / 57.8%	148.205 / 812.032	0.1187 / 0.3338	**Exploratory/Mixed**.
CIA	2-20	4.4% / 44.4%	97.025 / 54.537	0.1614 / 0.1865	Mixed/depends on timepoint.
SCL-GSI	2-18	4.4% / 40.0%	50.523 / 67.460	0.0528 / 0.0780	Mixed/depends on timepoint.
SCL90-DS	2-18	4.4% / 40.0%	60.140 / 52.991	0.0746 / 0.0857	Mixed/depends on timepoint.
EDS-TOT	3-18	6.7% / 40.0%	1.571 / 33.392	0.1909 / 0.9626	Mixed/Exploratory.
Y-BOCS	1-9	2.2% / 20.0%	68.902 / 74.121	0.4879 / 0.4666	Exploratory/limited by small N.

Note: T0 = baseline; T1 = 6-month follow-up. Missing n indicates the number of missing observations at each timepoint. Percentage of missing data is calculated relative to the total sample at each assessment. MissForest out-of-bag mean squared error (OOB-MSE) reflects prediction error during random forest–based imputation, with higher values indicating lower imputation accuracy. missRanger proportion of variance unexplained (PVU) represents the fraction of unexplained variance after imputation, with higher values indicating reduced reliability. Variables were classified a priori according to the extent of missingness and imputation quality as Exploratory (high missingness and/or high imputation error), Mixed (acceptable imputation quality at one timepoint but limited reliability at the other), or Exploratory/limited when constrained by small effective sample size. Results for variables with high missingness at T1 (>40%) should be interpreted cautiously and considered hypothesis-generating rather than confirmatory.
